# The value of prognostic clinical data in Bell’s palsy

**DOI:** 10.1016/S1808-8694(15)31198-8

**Published:** 2015-10-20

**Authors:** Cristiane A. Kasse, Oswaldo Laércio M. Cruz, Fernando D. Leonhardt, José Ricardo G. Testa, Ricardo G. Ferri, Érika Y. Viertler

**Affiliations:** 1 Full Professor, Affiliate Professor, Discipline of Pediatric Otorhinolaryngology, UNIFESP.; 2 Master studies in Otorhinolaryngology under course, Federal University of Sao Paulo, Ph.D., Discipline of Pediatric Otorhinolaryngology, Federal University of Sao Paulo and Sector of Otology.; 3 Ph.D. in Otorhinolaryngology, UNIFESP, Full Professor, Discipline of Pediatric Otorhinolaryngology, Federal University of Sao Paulo and Sector of Otology.; 4 Master in Otorhinolaryngology, Federal University of Sao Paulo.; 5 Ph.D. studies in Otorhinolaryngology under course, Federal University of Sao Paulo.; 6 Master in Otorhinolaryngology, Federal University of Sao Paulo. Post-graduate studies under course.

**Keywords:** Bell’s palsy, prognosis, clinical data, Hilger test

## Abstract

Electroneurography (ENoG) and clinical staging are currently the methods of choice to indicate prognosis in Bell’s palsy, although ENoG is an electrophysiological test not universally available. **Aim**: Identify other options of prognostic evaluation based upon clinical aspects and minimal electrical stimulation test allowing prognostic measurement in almost any circumstances. **Study design:** historic cohort. **Material and Method**: Chart review of 1,521 cases of IPFP, analyzing the following clinical aspects: gender, age, paralyzed side, installation mode, previous symptoms, associated symptoms and minimal electrical stimulation test (Hilger test) and its statistical correlation to facial palsy evolution after 6 months. **Results**: Data indicated that patients above 60 years old had worse prognosis in comparison with patients under 30 years old. A progressive mode of paralysis installation, absence of previous symptoms, concomitant vertigo and response superior to 3.5 mA at minimum electrical stimulation test were also related to worse prognosis. On the other hand, the absence of concomitant symptoms, diminished tearing and sudden onset were related to better prognosis. **Conclusion**: Clinical factors and Hilger’s test can accurately indicate the prognosis in cases of Bell’s palsy when ENoG is not available.

## INTRODUCTION

Bell’s palsy or idiopathic peripheral facial palsy is a relatively frequent disease all over the world. It affects all age ranges, without gender preference, with complete recovery in most cases. However, a considerable portion of the patients maintains permanent functional deficit causing psychological, social and professional abnormalities.

ENT examination, clinical history and otoneurological assessment should be carefully made and followed by complementary exams such as audiological assessment, imaging exams and biochemical analyses. These measures allow precise etiological diagnosis, leading to specific therapeutic indication. Normally, clinical information is directed to the attempt to define an etiological diagnosis, and the prognosis of palsy is defined by electrical tests, especially electroneuronography (ENoG). Even though it is a very important method to indicate the prognosis of facial palsy, EnoG is not available in all centers, especially in emergency centers. Conversely, basic clinical data are routinely obtained under any circumstances. Thus, it would be important to know the value of these clinical data in relation to disease prognosis. Factors such as gender, age, side of palsy, onset, previous symptoms, and associated symptoms most probably influence the progression of the cases. In addition, the test of minimum electrical stimulation (Hilger test) is a simple test to conduct and available in most centers of Otorhinolaryngology.

The present study aimed at statistically correlating clinical data and the results of Hilger test with level of final recovery of facial function in cases of Bell’s palsy, trying to identify which factors would have more prognostic value.

## MATERIAL AND METHOD

Cohort retrospective study analyzing 2,660 patients with Bell’s palsy followed up in the ambulatory of Otology, between 1986 and 2001. Out of the total, 1,521 patients formed the sample, complying with the following inclusion criteria: clinical and ENT assessment with classification of palsy according to House-Brackman classification^1^ in the first visit and after 6 months of progression; electrical stimulation test (Hilger)^2^ conducted in the first assessment, patients that were not submitted to clinical treatment with corticoids for clinical reasons (diabetes, decompensated blood hypertension, glaucoma, etc) or simply no compliance to medication.

The cases with poor initial progression that were referred to surgery were maintained in the assessment and their data were considered as indicative of poor prognosis.

The statistical analysis was performed with chi-square test, with level of significance of 5%. If the observed value (OV) was greater or equal to the critical value (CV), there was significant correlation.

## RESULTS

Out of 1,521 patients, 58.8% (894) were female patients and 41.2% (627) were male patients. Gender did not present any statistical difference concerning final grade of palsy (OV: 0.02, CV: 3.84).

**Graph 1:**
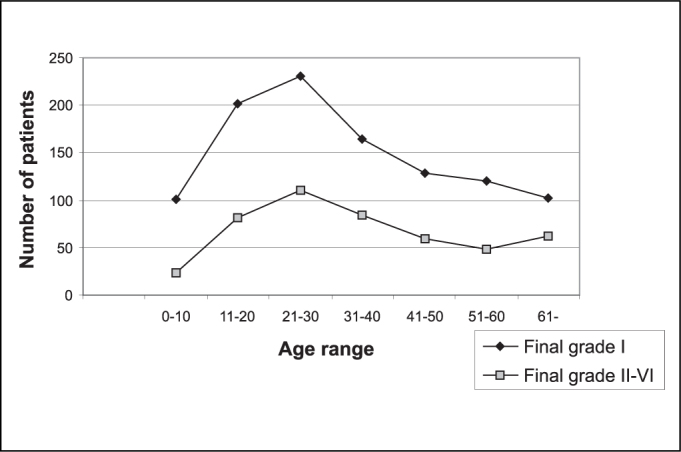
Correlation between age range and final grade of facial palsy. We could observe that the curves of favorable prognosis were parallel, with peak of incidence in the age range 21 to 30 years.

We detected higher incidence of palsy between ages 11 and 31 years, and the smallest incidence was detected in the extreme age ranges: between 0 and 10 years and over the age of 61 years (Table 1). As to progression, we observed that most of the patients (69%) progressed to final staging as grade I. The progression was proportional in all age ranges, as observed in Graph 1. However, there was statistically significant difference between final grade of facial palsy between the patients aged 0 to 10 years and patients with ages over 61 years, with higher incidence of final grade I in the first group and higher incidences of grades II-VI in the second group (OV:16.4 and CV 12.59). The other age ranges did not present significant difference concerning final grade of palsy.

**Table 1:** Correlation between final grade of facial palsy and age range.

Age range	Final grade I	%	Final grade II-VI	%	Total	%
0-10	101	9,6	24	5,1	125	8,2
11-20	201	19,2	81	17,3	282	18,6
21-30	231	22,1	110	23,5	341	22,5
31-40	164	15,7	84	17,9	248	16,4
41-50	128	12,2	60	12,8	188	12,4
51-60	120	11,5	48	10,2	168	11,1
61-	102	9,7	62	13,2	164	10,8

Total	1047	100	469	100	1516	

Chi-square test: observed value of 16.4 and critical value of 12.59 - there was positive correlation. The age range below 10 years presented higher incidence of final grade I whereas the age range over 61 years presented higher incidence of final grade II-VI.

As to affected side, 52.1% (792) of the patients presented right facial palsy and 47.9% (729) had left palsy, and there was no correlation between the palsy side and final staging (OV 0.10 and CV 3.84).

As to data related to previous symptoms, the incidence of patients without previous symptoms was 26.2%; earache complaint was found in 23.5%; nervousness (stress) in 19%, facial paresthesia in 15.8%. headache in 8.9%, aphthae in 4.6%, and other symptoms in 1.9%. There was a significant correlation between presence and absence of previous symptoms and final grade of palsy, and the absence of symptoms was statistically related with higher incidence of final grade II-VI (poor progression) (OV 18.09% and CV 12.59%). Individual analysis of previous symptoms did not present correlation with final grade of palsy.

As to concomitant symptoms, 21.5% of the patients had no symptoms, and there was hyper lachrymation in 23.8%, dysgeosia in 19.5%, hypo lachrymation in 10.8%, tinnitus in 9.4%, vertigo in 8.6% and other symptoms in 6.4%. There was significant correlation (OV 101.13, CV 14.07) between symptoms and final grade of palsy, and the absence of concomitant symptoms and hyper lachrymation were related to final grade I, whereas vertigo was more associated with final grade II-VI. Dysgeosia, hypo lachrymation and hearing loss did not present any correlation with progression.

Hilger test was performed in 1,292 patients. The test demonstrated a significant correlation with prognosis of the disease, and the patients with unexcitability presented higher incidence of final grade II-VI palsy. Among the cases that presented complete resolution, only 1.5% had compatible result with poor prognosis (different thresholds of excitability over 3.5mA or absence of excitability), whereas among patients with poor progression, 32% presented results that were considered to be unfavorable to the test, with statistically significant difference (OV 260.94, CV 3.84).

The sudden onset of palsy presented higher incidence (72.5%) than progressive onset (27.5%). Out of all patients that progressed to final grade I, 86.2% presented sudden onset facial palsy, whereas in the group of patients with final evolution to grades II-VI, this percentage was only 42.2% (Table 3). Thus, there was statistically significant difference in relation to type of palsy and prognosis, and progressive onset was related to unfavorable prognosis (OV:313.3, CV: 3.84).

**Table 3:** Correlation between final grade of facial palsy and type of onset.

Onset	Final grade I	%	Final grade II-VI	%	Total	%
Acute	901	86,2	201	42,2	1102	72,5
Progressive	144	13,8	275	57,8	419	27,5

Total	1045	100	476	100	1521	100

Chi-square test: observed value of 313.3, critical value of 3.84. There was significant correlation between onset of the disease and prognosis. Patients with sudden onset presented favorable diagnosis, whereas the others presented progressive onset and unfavorable prognosis.

## DISCUSSION

The present study revealed the incidence of peripheral facial palsy to be higher in women than in men, in a proportion of 1.44:1, without any statistically significant difference. In the literature, however, data are controversial. Adour et al ^3^, assessed 1,048 patients and did not observe any difference among genders, but the frequency was twice higher in female patients aged 10 to 19 years, and 1.5 times higher in male patients aged over 40 years. Devriese et al.^4^ did not find predominance between genders in their study with 1,293 cases. In the present study, there was no association between gender and grade of functional recovery, suggesting that this factor does not influence the prognosis of the disease.

**Table 2:** Relation between Hilger test and final grade of facial palsy.

Hilger	Final grade I	%	Final grade II-VI	%	Total	%
Excitable	873	98,5	276	68,0	1149	88,9
Unexcitable	13	1,5	130	32,0	143	11,1

Total	886	100	406	100	1292	

Chi-square test: observed value or 206.94, critical value of 3.84. There was significant correlation between Hilger test and the prognosis. The group with unexcitable Hilger presented higher incidence of unfavorable grade.

As to age, we observed that the highest number of Bell’s palsy cases occurred in the age range 21 to 30 years, corresponding to 22.5% of the total of patients. The lowest incidence was defined in extreme ranges (0 and 10 years and over 61 years). The prognosis proved to be more favorable in the younger age ranges, especially below 10 years, whereas patients over 61 years presented higher incidence of final grades II-VI. Katusic et al.^5^ observed higher rate of incomplete recovery in the age range above 55 years and Smith^6^, in 1988, in patients over the age of 60 years. Danielidis ^7^ related age as an important factor to determine the prognosis of the patient.

In our study, there was no predominance related to the affected side, and we did not observe any correlation between paralyzed side and recovery of palsy. We found only 1 case of bilateral facial palsy, which corresponded to 0.08% of the total, which is in accordance with the incidence below 1% reported by Gregg^8^, Leibowitz^9^, Adour^3^ and Yanagihara^10^.

Considering the most predominant previous symptoms, otalgia (23.5%), stress (19.0%), paresthesia (15.8%), headache (8.9%) and aphtha (4.6%) were the most frequent ones in our sample, whereas 26.2% did not present any prodromic signs or symptoms. May^11^ found otalgia in 50% of the patients, paresthesia in 40% and viral prodrome in 60%. Adour^3^ reported 20% viral prodrome, 10% headache and 32% facial paresthesia in the study with 515 patients. According to Fortes and Rego^12^ and Furuta^13^, insult to the nerve could be resultant from the reactivation of the latent nerve located in axons and nervous endings, triggering inflammation and infection in adjacent cells after a trauma, exposure to aggressive environmental factors or metabolic and/or emotional affections. Riskalla^14^ reported that in most of the idiopathic peripheral facial palsy cases, there was presence of stress before the onset of the disease. In this study, the patients without previous symptoms had higher incidence of final grade II-IV palsy. Conversely, the presence of previous paresthesia was correlated with good prognosis. The other symptoms proved to be indifferent concerning the progression.

As to concomitant symptoms to facial palsy, hyper lachrymation was the most frequent complaint (23.8%), followed by dysgeosia (19.5%) and hypo lachrymation (10.8%). We observed that absence of symptoms and hypo lachrymation were related with favorable prognosis, whereas vertigo was associated with final grade II-VI. Adour^3^, however, presented contrary results correlating unfavorable progression with presence of dysgeosia and hypo lachrymation. May^11^ agreed that hypo lachrymation would be an important clinical sign related with unfavorable prognosis. Other authors such as Hauser et al.^15^, Katusic et al.^5^ and Smith et al.^6^ did not identify statistically significant differences between prognosis and incidence of concomitant signs and symptoms in cases of idiopathic peripheral facial palsy. These statistical differences concerning prognosis and symptoms can be in part attributed to total number of studied patients and adopted methodology. Samples over 1,000 patients obviously present more consistent statistical data.

As to onset, we detected sudden onset in 72.5% of the cases and progressive onset in 27.5%, in agreement with Davidson^16^. Patients that progressed with final grade I presented 86.2% of sudden onset, whereas 57.8% of the patients that progressed unfavorably presented progressive onset, with statistically significant difference. Thus, sudden onset is indicative of better prognosis and progressive onset indicates poor evolution.

As to Hilger test, cases that presented complete resolution had 1.5% of compatible result with poor prognosis (difference between excitability thresholds over 3.5mA or absence of excitability), whereas among other patients with poor progression, 32% presented results that were unfavorable, resulting in statistically significant difference. Unfavorable results were considered those that had difference of minimum excitability greater than 3.5mA, as suggested by authors such as Campbell^17^ and Laumans and Jongkees^18^. The prognostic value is reported as having sensitivity between 85 and 95% by Jackson^19^, Lewis et al.^20^ who reported that the test proved to be efficient, economic and easy to handle, which is a method for prognostic screening in cases of peripheral facial palsy, which is in agreement with our results. Even though without the specificity of EnoG, this test can, together with other pieces of information, be used to estimate the prognosis.

## CONCLUSION

Data obtained from assessment of 1,521 patients with Bell’s palsy in our service indicated that younger patients (especially those below 20 years) presented better prognosis compared to older patients (over 61 years); absence of previous symptoms before onset of palsy was related to poor prognosis; absence of concomitant symptoms and hypo lachrymation during palsy were related with good prognosis, whereas vertigo concomitant with palsy indicated unfavorable prognosis; sudden onset of palsy was related with good prognosis whereas progressive onset indicated poor prognosis; favorable result of Hilger test was related with good prognosis.
